# The effects of enzymatically treated soybean meal on growth performance and intestinal structure, barrier integrity, inflammation, oxidative status, and volatile fatty acid production of nursery pigs

**DOI:** 10.1093/tas/txaa170

**Published:** 2020-09-10

**Authors:** Leigh A Ruckman, Amy L Petry, Stacie A Gould, Brian J Kerr, John F Patience

**Affiliations:** 1 Department of Animal Science, Iowa State University, Ames; 2 National Laboratory for Agriculture and the Environment, USDA-ARS, Ames, IA

**Keywords:** cytokines, gut health, health, oxidative status, swine, weaned pig

## Abstract

The objective of this experiment was to determine the impact of diets containing increasing amounts of enzymatically treated soybean meal (ESBM) but decreasing amounts of soybean meal (SBM) on growth performance, intestinal structure, and barrier integrity, inflammation, and oxidative status in weaned pigs. A total of 480 pigs [6.3 ± 1.2 kg body weight (BW)] were blocked by initial BW and pens (*n* = 12 per treatment) were randomly allotted to one of four dietary treatments. Diets were fed in three phases (days 0–14, 14–28, and 28–35) over a 35-d period. The four dietary treatments consisted of a negative control diet (NC): the NC with 7.0% ESBM (ESBM1), the NC with 14.0% ESBM (ESBM2), and the NC with 21.0% ESBM (ESBM3). Soybean meal was reduced proportionately in each treatment. In phase 2, ESBM inclusion was decreased by 50% (3.5%, 7.0%, and 10.5% ESBM, respectively); phase 3 was a common diet and contained no ESBM. Fecal score was visually ranked weekly using a four-point scale. Intestinal tissue, digesta, and blood samples were collected from 48 pigs (1 per pen) on day 10. Data were analyzed using PROC MIXED of SAS (9.4) with pen as the experimental unit; diet and block were considered fixed effects. Linear and quadratic contrasts were used to determine the effect of increasing ESBM. Overall, ESBM2 and ESBM3 decreased final BW, average daily gain, and average daily feed intake compared to NC and ESBM1 (diet, *P* < 0.05; linear, *P* < 0.05). Overall fecal score (diet, *P* < 0.05) and fecal dry matter (*P* < 0.05) were improved by feeding ESBM diets compared to NC. Volatile fatty acid (VFA) concentration of acetate, propionate, butyrate, and total VFA in ileal contents increased as ESBM inclusion increased (*P* < 0.05). Colonic VFA concentration was not impacted (*P* > 0.10). Total antioxidant capacity was increased by ESBM (*P* < 0.05). The concentration of mucosal interleukin-4 increased as the inclusion of ESBM increased (linear, *P* < 0.05). Messenger ribonucleic acid abundance of *occludin* and *zonula-occludens-1* in ileal tissue was increased by ESBM1 or ESBM2 (*P* < 0.05). In conclusion, increasing the dietary levels of ESBM over 7% had a negative impact on nursery pig performance, but ESBM positively impacted fecal score. Feeding ESBM improved oxidative status and intestinal barrier integrity while increasing ileal VFA production but had minimal impact on intestinal inflammation or morphology. Further research is needed to determine the optimal inclusion level of ESBM.

## INTRODUCTION

The transition at weaning exposes pigs to multiple new stressors, leaving them susceptible to low feed intake and reduced growth, gastrointestinal tract (GIT) disorders, and impaired intestinal function and integrity (Lallès et al., 2004; Moeser et al., 2007; Li et al., 2020). Furthermore, a suppressed immune system and still-developing GIT can increase weaned pigs’ vulnerability to pathogens and enteric disease (de Lange et al., 2010; Becker et al., 2020). These issues have generated greater interest in feeding strategies that will positively affect intestinal health and function of weaned pigs.

Soybean meal (SBM) is frequently the main protein source in swine diets. The processing of raw soybeans into SBM includes heat treatment, which inactivates the majority of trypsin inhibitor and urease (Herkelman et al., 1992; Woyengo et al., 2017). However, the concentration of other antinutritional factors (ANF) in SBM may be high enough to negatively impact the growth and intestinal health of young pigs (Li et al., 1991; Yang et al., 2007). Glycinin and β-conglycinin, the main antigenic proteins found in SBM, cause a hypersensitive immune response in the GIT of weaned pigs, resulting in abnormal morphology of the small intestine and reduced absorptive capacity (Li et al., 1991). Furthermore, the nondigestible oligosaccharides (NDO) in soybeans (specifically stachyose and raffinose) can cause diarrhea while reducing growth (Zhang et al., 2003). These dietary components limit the use of SBM in early nursery diets, leading to greater use of protein sources of animal origin that are highly digestible but also quite expensive (Min et al., 2009). Thus, further processing methods for SBM have been developed to diminish the concentration of ANF.

Enzymatically treated SBM (ESBM) is produced by treating SBM with proprietary blends of microbial enzymes (typically including proteases and carbohydrases) for several hours (Goebel and Stein, 2011). The resulting ingredient has reduced levels of several ANF and improved digestibility of amino acids and crude protein (CP) compared to conventional SBM (Cervantes-Pahm and Stein, 2010; Ma et al., 2019b). The ESBM is considered a high-quality protein source for weaned pigs as it has been reported that replacing conventional SBM with ESBM in nursery pig diets can improve gain and feed conversion (Zhu et al., 1998; Zhou et al., 2011). However, the impact of ESBM on the intestinal health and function of weaned pigs is still largely unknown.

The objective of this experiment was to determine the impact of diets in which ESBM replaced increasing amounts of SBM on growth performance, intestinal structure and barrier integrity, inflammation, and oxidative status in newly weaned pigs. It was hypothesized that replacing conventional SBM with ESBM would improve the growth performance of pigs while beneficially modulating markers of intestinal structure and barrier integrity, immune status, and oxidative status.

## MATERIALS AND METHODS

All experimental procedures adhered to the principles for the ethical and humane use of animals for research according to the Guide for the Care and Use of Agricultural Animals in Research and Teaching (FASS, 2010) and were approved by the Iowa State University Animal Care and Use Committee (IACUC-19–073).

### Animals, Housing and Experimental Design

A total of 480 pigs [6.34 ± 1.18 kg body weight (BW); L337 × Camborough, PIC, Hendersonville, TN] were weaned at approximately 21 d and transported to the Iowa State University Swine Nutrition Farm (Ames, IA). Upon arrival, pigs were ear-tagged, weighed individually, and vaccinated against K88+ and F18 *Escherichia coli* via a water-delivered vaccine (Entero Vac and Edema Vac, Arko Laboratories, Jewell, IA). Pigs were blocked by initial BW and pens were randomly assigned to one of four dietary treatments. Each pen housed 10 pigs and there were 12 pens per treatment. Sexes were not separated by pen, but similar numbers of barrows and gilts were assigned to each pen within a block. Each pen (1.2 m × 2.4 m) had a wire mesh floor and was equipped with a four-space dry self-feeder and two nipple waterers to provide ad libitum access to feed and water.

### Dietary Treatments and Feeding

Experimental diets were fed in three phases over 35 d (Tables 1 and 2). Phase 1 was fed from day 0 to day 14, phase 2 was fed from day 14 to day 28, and phase 3 was fed from day 28 to day 35. The dietary treatments consisted of a negative control diet (NC) with no ESBM, NC diet with 7.0% ESBM (HP 300, Hamlet Protein Inc., Findlay, OH) and reduced SBM (ESBM1), NC diet with 14.0% ESBM and a larger reduction of SBM (ESBM2), and NC with 21.0% ESBM and the elimination of SBM (ESBM3). In phase 2, the inclusion of ESBM in the ESBM1, ESBM2, and ESBM3 diets was decreased by 50% (3.5%, 7.0%, and 10.5% ESBM, respectively). The phase 3 diet was a common diet and contained no ESBM. The NC diets contained 25.75% SBM in phase 1, 28.84% in phase 2, and 31.41% in phase 3. The ESBM1, ESBM2, and ESBM3 diets contained 16.97%, 8.35%, and 0% SBM in phase 1 and 24.40%, 19.95%, and 15.46% SBM in phase 2, respectively.

**Table 1.** Ingredient and nutrient composition of experimental diets (as-fed basis): phase 1^*a*,*b*^

	Phase 1			
Diet^*c*^	NC	ESBM1	ESBM2	ESBM3
Ingredient composition, %				
Corn	36.49	38.27	39.91	41.27
SBM^*d*^	25.75	16.97	8.35	–
Oat groats	12.50	12.50	12.50	12.50
Whey permeate	15.00	15.00	15.00	15.00
Milk casein	3.00	3.00	3.00	3.00
ESBM^*e*^	–	7.00	14.00	21.00
Corn oil	3.00	3.00	3.00	3.00
L-lysine HCl	0.51	0.51	0.50	0.49
DL-methionine	0.28	0.28	0.27	0.27
L-threonine	0.27	0.26	0.25	0.24
L-tryptophan	0.02	0.03	0.03	0.04
L-valine	0.10	0.08	0.05	0.03
Monocalcium phosphate 21%	0.57	0.57	0.57	0.57
Limestone	1.44	1.47	1.50	1.52
Salt	0.58	0.58	0.58	0.58
Vitamin premix^*f*^	0.24	0.24	0.24	0.24
Trace mineral premix^*g*^	0.20	0.20	0.20	0.20
Phytase^*h*^	0.05	0.05	0.05	0.05
Calculated nutrients				
Total Lys, %	1.53	1.53	1.53	1.53
SID Lys, %	1.40	1.40	1.40	1.40
SID TSAA:Lys	0.58	0.58	0.58	0.58
SID Thr:Lys	0.61	0.61	0.61	0.61
SID Trp:Lys	0.18	0.18	0.18	0.18
NDF, %	6.65	6.42	6.19	5.96
Ca, %	0.85	0.85	0.85	0.85
STTD P, %	0.43	0.43	0.43	0.43
ME, Mcal/kg	3.41	3.42	3.44	3.45
NE, Mcal/kg	2.46	2.50	2.54	2.58
Analyzed nutrients				
DM, %	88.83	89.91	90.99	91.06
Ash, %	5.74	5.78	6.17	6.07
CP, %	19.91	19.57	20.23	20.86
aEE, %	5.72	5.76	5.97	6.04
Total Lys, %	1.47	1.34	1.54	1.34
SID Lys, %	1.35	1.23	1.41	1.23
SID TSAA, %	0.71	0.76	0.74	0.81
SID TSAA:Lys	0.51	0.58	0.55	0.65
SID Thr, %	0.83	0.85	0.83	0.85
SID Thr:Lys	0.59	0.65	0.61	0.68
SID Trp, %	0.22	0.23	0.22	0.23
SID Trp:Lys	0.16	0.18	0.16	0.18
SID Ile, %	0.74	0.78	0.80	0.83
SID Ile:Lys	0.53	0.60	0.59	0.66
SID Val, %	0.86	0.95	0.93	0.95
SID Val:Lys	0.61	0.73	0.69	0.76
GE, Mcal/kg	3.91	3.94	3.94	3.99
Ca, %	0.70	0.85	0.91	0.87
Total P, %	0.56	0.55	0.57	0.56
WHC, mL/g of DM	1.08	1.14	1.22	1.40

ME, metabolizable energy; NDF, neutral detergent fiber; NE, net energy; STTD, standardized total tract digestible; TSAA, total sulfur amino acids (Met + Cys).

^*a*^Phase 1 was fed from day 0 to day 14.

^*b*^All diets were formulated to meet or exceed nutrient requirements of the pigs (NRC, 2012).

^*c*^NC: negative control, 0% ESBM; ESBM1: 7% ESBM; ESBM2: 14% ESBM; ESBM3: 21% ESBM.

^*d*^Dehulled, solvent-extracted soybean meal.

^*e*^HP 300 (Hamlet Protein Inc., Findlay, OH).

^*f*^The vitamin premix provided per kilogram of complete diet: 7,350 IU vitamin A, 840 IU vitamin D3, 60 IU vitamin E, 3.6 mg vitamin K, 13.2 mg riboflavin, 67.2 mg niacin, 32.4 mg pantothenic acid, and 60 μg vitamin B_12_.

^*g*^The trace mineral premix provided per kilogram of complete diet: 160 ppm Fe as FeSO_4_, 160 ppm Zn as ZnSO_4_, 9 ppm Mn as MnSO_4_, 12 ppm Cu as CuSO_4_, 0.3 ppm I as C_2_H_10_I_2_N_2_ or KIO_3_, and 0.3 ppm Se as Na_2_SeO_4_ or Na_2_SeO_3_.

^*h*^Quantum Blue 5 G (AB Vista Feed Ingredients, Marlborough, Wiltshire, UK) was added at 0.05% for 2,500 FTU/kg.

**Table 2.** Ingredient and nutrient composition of experimental diets (as-fed basis): phase 2 and 3^*a*,*b*^

	Phase 2				Phase 3
Diet^*c*^	NC	ESBM1	ESBM2	ESBM3	NC
Ingredient composition, %					
Corn	50.81	51.75	52.69	53.68	61.81
SBM^*d*^	28.84	24.40	19.95	15.46	31.41
Oat groats	5.00	5.00	5.00	5.00	–
Whey permeate	7.50	7.50	7.50	7.50	–
Milk casein	0.85	0.85	0.85	0.85	–
ESBM^*e*^	–	3.50	7.00	10.50	–
Corn oil	3.00	3.00	3.00	3.00	3.00
L-lysine HCl	0.50	0.50	0.50	0.50	0.42
DL-methionine	0.26	0.26	0.26	0.26	0.22
L-threonine	0.25	0.24	0.24	0.23	0.19
L-tryptophan	0.02	0.02	0.02	0.02	–
L-valine	0.12	0.11	0.10	0.09	0.05
Monocalcium phosphate 21%	0.52	0.52	0.52	0.52	0.57
Limestone	1.29	1.30	1.32	1.33	1.23
Salt	0.56	0.56	0.56	0.56	0.61
Vitamin premix^*f*^	0.24	0.24	0.24	0.24	0.24
Trace mineral premix^*g*^	0.20	0.20	0.20	0.20	0.20
Phytase^*h*^	0.05	0.05	0.05	0.05	0.05
Calculated nutrients					
Total Lys, %	1.43	1.43	1.43	1.43	1.37
SID Lys, %	1.30	1.30	1.30	1.30	1.23
SID TSAA:Lys	0.58	0.58	0.58	0.58	0.58
SID Thr:Lys	0.61	0.61	0.61	0.61	0.60
SID Trp:Lys	0.18	0.18	0.18	0.18	0.18
NDF, %	7.48	7.37	7.25	7.14	8.21
Ca, %	0.75	0.75	0.75	0.75	0.70
STTD P, %	0.38	0.38	0.38	0.38	0.35
ME, Mcal/kg	3.40	3.41	3.42	3.42	3.40
NE, Mcal/kg	2.48	2.50	2.52	2.54	2.50
Analyzed nutrients					
DM, %	88.66	88.96	89.35	89.88	87.47
Ash, %	5.49	5.67	5.62	5.66	5.28
CP, %	18.64	18.57	19.32	19.07	17.44
aEE, %	6.15	6.15	6.08	5.94	6.41
Total Lys, %	1.43	1.43	1.50	1.47	1.39
SID Lys, %	1.30	1.30	1.37	1.33	1.24
SID TSAA, %	0.63	0.73	0.79	0.76	0.75
SID TSAA:Lys	0.48	0.56	0.58	0.57	0.60
SID Thr, %	0.86	0.79	0.75	0.81	0.80
SID Thr:Lys	0.66	0.61	0.55	0.61	0.65
SID Trp, %	0.20	0.20	0.20	0.21	0.21
SID Trp:Lys	0.15	0.15	0.15	0.16	0.17
SID Ile, %	0.67	0.69	0.73	0.70	0.69
SID Ile:Lys	0.52	0.53	0.53	0.53	0.56
SID Val, %	0.84	0.83	0.88	0.89	0.87
SID Val:Lys	0.65	0.64	0.64	0.67	0.70
GE, Mcal/kg	3.90	3.91	3.94	3.94	3.86
Ca, %	0.79	0.76	0.81	0.75	0.72
Total P, %	0.50	0.49	0.51	0.50	0.49
WHC, mL/g of DM	1.40	1.49	1.44	1.46	1.21

ME, metabolizable energy; NDF, neutral detergent fiber; NE, net energy; STTD, standardized total tract digestible; TSAA, total sulfur amino acids (Met + Cys).

^*a*^Phase 2 was fed from day 14 to day 28 and phase 3 was fed from day 28 to day 35. Phase 3 was a common diet across all treatments.

^*b*^All diets were formulated to meet or exceed nutrient requirements of the pigs (NRC, 2012).

^*c*^NC: negative control, 0% ESBM; ESBM1: 3.5% ESBM; ESBM2: 7% ESBM; ESBM3: 10.5% ESBM

^*d*^Dehulled, solvent-extracted soybean meal

^*e*^HP 300 (Hamlet Protein Inc., Findlay, OH).

^*f*^The vitamin premix provided per kilogram of complete diet: 7,350 IU vitamin A, 840 IU vitamin D3, 60 IU vitamin E, 3.6 mg vitamin K, 13.2 mg riboflavin, 67.2 mg niacin, 32.4 mg pantothenic acid, and 60 μg vitamin B_12_.

^*g*^The trace mineral premix provided per kilogram of complete diet: 160 ppm Fe as FeSO_4_, 160 ppm Zn as ZnSO_4_, 9 ppm Mn as MnSO_4_, 12 ppm Cu as CuSO_4_, 0.3 ppm I as C_2_H_10_I_2_N_2_ or KIO_3_, and 0.3 ppm Se as Na_2_SeO_4_ or Na_2_SeO_3_.

^*h*^Quantum Blue 5 G (AB Vista Feed Ingredients, Marlborough, Wiltshire, UK) was added at 0.05% for 2,500 FTU/kg.

All diets were formulated to meet or exceed the pigs’ nutrient requirements for each phase (NRC, 2012). Because the experiment was designed to evaluate a specific ingredient, it was important to minimize changes in the levels of all other ingredients in the formulation while, at the same time, as much as possible, also maintaining constant energy and nutrient levels; otherwise, the results of the study could be confounded. Therefore, the levels of most ingredients across treatments within phase were identical. The only exceptions were corn, SBM, and ESBM; very small differences in the levels of select synthetic amino acids and limestone were necessary to maintain constant calcium and SID amino acid concentrations. All diets were fed in mash form.

### Medical Treatments

When required, and according to the farm protocol, pigs were individually treated with ceftiofur (Excede, Zoetis, Florham Park, NJ); animals not responsive to medical treatment were removed from the study. Individual medical treatments were recorded by pen, day, and dosage. Under the direction of a veterinarian, sodium salicylate (Oral-Pro Sodium Salicylate Concentrate, Aurora Pharmaceutical, Northfield, MN; 78 mL of sodium salicylate concentrate per liter of water) was added to the water on days 13–17 and days 34–35 to treat lethargy and respiratory symptoms.

### Data and Sample Collection

Pigs were individually weighed on days 0, 14, 28, and 35 to determine average daily gain (ADG). Feed disappearance was also recorded on each weigh day to determine average daily feed intake (ADFI) and to calculate gain:feed (G:F). Weights and removal dates of pigs were recorded and ADG and ADFI were calculated according to pig days on test. Fecal consistency was scored by pen on days 7, 14, 21, 28, and 35 using the following categorical scale: 1 = solid, 2 = semisolid, 3 = semiliquid, and 4 = liquid. Fecal score was assessed independently by two people, and an average score of these two was recorded for each pen during each week.

Blood collection and necropsies were performed on day 10. This time point was selected so that sampling occurred during the peak of the adjustment period. This makes this the best time to evaluate gut adaptation to the weaning diet and the most likely time to observe treatment differences. One pig from each pen (12 per treatment) was selected, and blood samples were collected by jugular venipuncture into two 10-mL vacutainer tubes (Becton Dickinson, Franklin Lakes, NJ) to obtain plasma and serum. Plasma tubes were centrifuged at 1,000 × *g* for 10 min at 4 °C and serum tubes were centrifuged at 1,500 × *g* for 15 min at 4 °C before aliquots were harvested and stored at −80 **°**C.

Pigs were subsequently euthanized by captive bolt stunning followed by exsanguination. Ileal tissues were collected 10 cm proximal to the ileocecal junction. Digesta was collected and tissues were rinsed with phosphate-buffered solution (PBS) before both were snap-frozen in liquid nitrogen and stored at −80 **°**C. Two tissue samples were collected from the mid-ileum, rinsed with PBS, and fixed in 10% neutral buffered formalin for 24 h and then transferred to 70% ethanol. Digesta was collected from the mid-colon, snap-frozen in liquid nitrogen, and stored at −80 °C. The pH and temperature of ileal and colonic digesta were measured using a portable pH meter (pH 150 Meter Kit, Oakton Instruments, Vernon Hills, IL). Fresh ileal and colonic digesta samples were collected into tubes and stored on ice.

Feed samples were collected during the manufacturing of the diets. Multiple subsamples of each diet were collected throughout each batch of feed and homogenized before being stored at −20 °C. On days 11–13, fecal samples were collected via grab sampling from each pen and stored at −20 °C until further processing.

### Chemical Analysis

Diets were ground to 1 mm particle size (Variable Speed Digital ED-5 Wiley Mill; Thomas Scientific, Swedesboro, NJ), dried at 60 °C to a constant weight, and analyzed in duplicate for dry matter (DM; method 930.15; AOAC, 2007), ash (method 942.05; AOAC, 2007), acid hydrolyzed ether extract (aEE; method 2003.06; AOAC, 2007), and nitrogen (N; method 990.03; AOAC, 2007; TruMac; LECO Corp., St. Joseph, MI). Crude protein was calculated as N × 6.25 with ethylenediaminetetraacetate (EDTA; 9.56% N; determined to have 9.56 ± 0.03% N) used for standard calibration. An isoperibolic bomb calorimeter was used to determine gross energy (GE; model 6200; Parr Instrument Co., Moline, IL); benzoic acid (6318 kcal GE/kg; Parr Instrument Co., Moline, IL), determined to contain 6,323 ± 7 kcal GE/kg, was used as the calibration standard. Standardized ileal digestible (SID) amino acid levels of the diets were calculated using the assayed total amino acid concentration (Agricultural Experiment Station Chemical Laboratories, University of Missouri, Columbia, MO) and the ingredient’s SID coefficient for individual amino acids (NRC, 2012). Diets were analyzed for Ca and total P (Eurofins US, Des Moines, IA) using inductively coupled plasma optical emission spectrometry (AOAC 984.27, 927.02, 985.01, and 965.17). Fecal samples were thawed, homogenized, and then dried to a constant weight at 60 °C. Dried feces were ground using a mortar and pestle and analyzed in duplicate for DM (method 930.15; AOAC, 2007). The coefficient of variation (CV) threshold for repeating an assay was 1% for DM, ash, CP, GE, and fecal DM and 5% for aEE.

The concentration of volatile fatty acids (VFAs) was measured in ileal and colonic digesta in triplicate. Colonic digesta (1 g) was diluted with 5 mL of deionized water and mixed overnight on a rocking platform before centrifugation at 20,000 × *g* for 20 min at 4 °C. The supernatant (1 mL) was placed into a gas chromatography (GC) vial with 0.3 g of NaCl and 100 μL of phosphoric acid. Ileal digesta (2 g) was centrifuged at 20,000 × *g* for 20 min at 4 °C before the supernatant (1 mL) was placed into a new tube with 100 μL of phosphoric acid. The tubes were centrifuged at 4,000 × *g* for 10 min at 4 °C and the supernatant (1 mL) was placed into a GC vial with 0.3 g of NaCl. The prepared samples were frozen at −20 °C and sent to an external laboratory (USDA-ARS-MWA-NLAE, Ames, IA) for GC analysis (Agilent 7890A Gas Chromatograph, Agilent Technologies Inc., Wilmington, DE) using methods previously described by Kerr et al. (2015). The total VFA concentration is the sum of acetate, propionate, and butyrate concentration and is expressed as millimolars of VFA per liter of digesta. The molar proportions of VFA (%) were calculated using individual and total VFA concentration: [(mM VFA_individual_/L ÷ mM VFA_total_/L) × 100]. The CV threshold was less than 15% for all VFA analyses.

The water-holding capacity (WHC) of feed was measured in triplicate using a modified protocol from Giger-Reverdin (2000). Dried and ground feed (0.5 g) was soaked in 50 mL of deionized water for 24 h. The sample was filtered using a fritted funnel (40–60 µm porosity) for 1 h and the remaining wet sample was weighed. The WHC was calculated using the following equation (WHC = grams of retained water/grams of dry feed) and was expressed as milliliters of water per gram of DM. The CV threshold was less than 5% for WHC. The water-binding capacity (WBC) of ileal and colonic digesta was measured in triplicate using a modified protocol from Serena et al. (2008). Fresh digesta (2 g) was centrifuged at 10,000 × *g* for 20 min at 4 °C to separate the liquid and solid components. The liquid fraction was removed by suction immediately after centrifugation and again 12 h later. The solid fraction was weighed, and the WBC was calculated using the wet weight (WW) and dry weight (DW) of the digesta [WBC = (WW − DW)/DW].

### Oxidative Status, Lipopolysaccharide-Binding Protein, and Mucosal Cytokines

Ileal tissue samples (100 mg) were sonicated in 1 mL of buffer (RIPA buffer, Sigma-Aldrich, St. Louis, MO), containing 1 mM of EDTA disodium salt before centrifugation at 1,600 × *g* for 10 min at 4 °C. The total protein concentration of the resulting lysate was measured using a Pierce bicinchoninic acid (BCA) Protein Assay Kit (Thermo Fisher Scientific, Woltham, MA). Malondialdehyde (MDA) was measured in plasma and ileal tissue lysate using the colorimetric protocol of a thiobarbituric acid reactive substances (TBARS) kit (TBARS Assay Kit, Cayman Chemical Company, Ann Arbor, MI) as previously described (Armstrong and Browne, 1994; Yagi, 1998). The MDA concentration of ileal tissue lysate was expressed as micromolars of MDA per microgram of protein.

Total antioxidant capacity (TAC) was measured in plasma (diluted 1:10 with provided assay buffer) using a commercially available colorimetric assay (Antioxidant Assay Kit, Cayman Chemical Company, Ann Arbor, MI) as previously described (Miller et al., 1993). Lipopolysaccharide-binding protein (LBP) was measured in serum (diluted 1:300 with provided assay buffer) using a sandwich enzyme-linked immunosorbent assay (ELISA) kit (LBP various species ELISA kit, Hycult Biotech, Uden, The Netherlands). The CV threshold was less than 5% for total protein, MDA, and TAC values and less than 10% for LBP values.

Ileal mucosal homogenates were prepared using methods previously described by Becker et al. (2020). The homogenates were sent to a commercial laboratory (Eve Technologies Corporation, Calgary, AB, Canada) and analyzed for cytokines using a multiplex immunoassay that utilized laser bead technology. The assay included granulocyte-macrophage colony-stimulating factor (GM-CSF), interferon-γ (IFNγ), interleukin 1α (IL-1α), IL-1β, IL-1 receptor antagonist (IL-1RA), IL-2, IL-4, IL-6, IL-8, IL-10, IL-12, IL-18, and tumor necrosis factor-α (TNF-α).

### RNA Isolation and Real-Time Quantitative PCR

Ileal tissues (25–40 mg) were homogenized in a lysis buffer (RNeasy Plus Mini Kit, Qiagen, Carlsbad, CA) using the Qiagen Tissuelyser II (Germantown, MD) to isolate total RNA. The RNA was treated with a deoxyribonuclease enzyme to prevent genomic DNA contamination (DNA-free DNA removal kit, Invitrogen, Carlsbad, CA). A spectrophotometer (ND-100; NanoDrop Technologies Inc., Rockland, DE) was used to quantify RNA concentration and all samples had 260:280 nm ratios above 1.8. Complementary DNA (cDNA) was synthesized from isolated RNA (0.8 μg) using the QuantiTect Reverse Transcription Kit (Qiagen, Hilden, Germany) and all cDNA samples were diluted 10-fold with nuclease-free water.

Real-time quantitative polymerase chain reaction (RT-qPCR) was performed in triplicate using iQ SYBR Green Supermix (Bio-Rad Laboratories, Inc., Hercules, CA). The gene-specific primers (Table 3) were diluted to 10 µM with nuclease-free water. Each 20-µL reaction included 10 µL of SYBR Green Supermix, 1 µL of each forward and reverse primer, 3 µL of cDNA, and 5 µL of nuclease-free water. Each plate included a no-reverse transcriptase negative control and a pooled cDNA reference sample. An RT-qPCR detection system (iQ5; Bio-Rad Laboratories Inc.) was used to quantify SYBR Green fluorescence with the following cycling conditions: 5-min initial denaturation at 95 °C followed by 40 RT-qPCR cycles (95 °C for 30 s, 55 or 60 °C for 30 s, and 72 °C for 30 s) and a dissociation curve to verify the amplification of a single RT-qPCR product. Optical System Software (iQ5, version 2.0; Bio-Rad Laboratories Inc) was used to analyze amplification plots and cycle threshold (Ct) values for each reaction were obtained. The messenger RNA (mRNA) abundance was normalized to a reference gene (ribosomal protein L19) and the pooled sample. The 2^-ΔΔCT^ method (Livak and Schmittgen, 2001) was used to calculate fold change. The CV threshold was less than 5% for all RT-qPCR analysis.

**Table 3.** Primers used for RT-qPCR

Gene	Primer sequence, 5’→3’	Product size, base pair	GenBank accession	Annealing temperature, °C
*CLDN3*	F: TTGCATCCGAGACCAGTCC	85	NM_001160075	60
	R: AGCTGGGGAGGGTGACA			
*CLDN4*	F: CAACTGCGTGGATGATGAGA	140	NM_001161637	60
	R: CCAGGGGATTGTAGAAGTCG			
*OCLN*	F: TCGTCCAACGGGAAAGTGAA	95	NM_001163647	55
	R: ATCAGTGGAAGTTCCTGAACCA			
*ZO-1*	F: CTCTTGGCTTGCTATTCG	197	XM_003353439	55
	R: AGTCTTCCCTGCTCTTGC			
*RPL19*	F: AACTCCCGTCAGCAGATCC	147	AF_435591	55
	R: AGTACCCTTCCGCTTACCG			

F, forward primer; R, reverse primer; RPL19, ribosomal protein-L19.

### Intestinal Morphology

Ileal tissues (fixed in 70% ethanol) were embedded in paraffin wax, sectioned, stained with hematoxylin and eosin, and mounted on glass slides (Iowa State Veterinary Diagnostics Lab, Ames, IA). A DP80 Olympus Camera mounted on an OLYMPUS BX 53/43 microscope (Olympus Scientific, Waltham, MA) was used to take images of slides at 10× power. Ten villi and crypt pairs per ileal sample were measured using OLYMPUS CellSens Dimension 1.16 software. The ratio of villus height to crypt depth was calculated for each pair (V:C ratio).

### Statistical Analysis

Data were analyzed using the following mixed models. Model 1 assumed that residuals were normally distributed with an unstructured (UN) dependent covariance structure $[N(0,I\;UN\sigma_{e}^{2}$)]. The following mixed model was used to analyze data for fecal score and growth performance by phase.

$$\begin{array}{l} Model\;\;1:\;Y_{ijkl}=\mu +\tau_{i}+\rho_{k}+\tau_{i\;}\rho_{k}+a_{j}+e_{ijkl} \end{array}$$

where $Y_{ijkl}$ is the observed value for *l*th experimental unit (pen) within the *k*th period in the *j*th block within the *i*th level of diet; *µ* is the general mean; Undefined control sequence \bold is the fixed effect of the *i*th diet (*i* = NC, ESBM1, ESBM2, and ESBM3); Undefined control sequence \bold is the fixed effect of the *j*th block (*j* = 1–12); Undefined control sequence \bold is the fixed effect of period (*k* = 1–3); Undefined control sequence \bold is the interaction term of diet × period; and $e_{ijkl}$ is the associated variance as described by the model for $Y_{ijkl}$ (*l* = 1–48), assuming $e_{ijkl}∼N(0,I\;UN\;\sigma_{e}^{2}$), where Undefined control sequence \bold is the identity matrix.

Model 2 assumed that residuals were independent and normally distributed $[N(0,I\;\sigma_{e}^{2}$)]. The following mixed model was used to analyze all data except for fecal score and growth performance by phase.

$$\begin{array}{l} Model\;\;2:\;Y_{ijk}=\mu +\tau_{i}+a_{j}+e_{ijk} \end{array}$$

where $Y_{ijk}$ is the observed value for the *k*th experimental unit (pen) within the *j*th block in the *i*th level of diet; *μ* is the general mean; Undefined control sequence \bold is the fixed effect of the *i*th diet (*i* = NC, ESBM1, ESBM2, and ESBM3); Undefined control sequence \bold is the fixed effect of the *j*th block (*j* = 1–12); and $e_{ijk}$ is the associated variance as described by the model for $Y_{ijk}$ (*k* = 1–48), assuming $e_{ijk}∼N(0,I\;\sigma_{e}^{2}$), where Undefined control sequence \bold is the identity matrix.

Normality and homogeneity of the studentized residuals from the reported models were verified using the UNIVARIATE procedure of SAS 9.4 (SAS Inst., Cary, NC). Statistical outliers, defined as being greater than 3 SDs from the mean, were identified and removed from the analysis. All data and models were analyzed using the MIXED procedure. The UN covariance structure was selected as the best fit for model 1 according to Bayesian Information Criterion for all dependent variables. Linear and quadratic orthogonal polynomial contrasts were applied to determine the effects of increasing levels of ESBM. Fisher’s least significant difference test was used to separate least square means, and differences were considered significant if *P* < 0.05 and trends if 0.05 ≥ *P* < 0.10.

## RESULTS

### Health and Fecal Score

The total removal rate of pigs, including mortalities and removals due to illness, was 1.3%. Pigs fed NC and ESBM2 required more medical treatments than pigs fed ESBM1, and ESBM3 was the intermediate between them (diet, *P* = 0.038; Table 4). Feeding NC increased the overall fecal score compared to ESBM1, ESBM2, and ESBM3 (diet, *P* = 0.003; Table 5). No diet × period interactions were observed for fecal score (*P* > 0.10).

**Table 4.** The effect of increasing ESBM on the number of medical treatments^*a*,*b*,c^

						*P*-value		
Item	NC	ESBM1	ESBM2	ESBM3	SEM	Diet	Linear	Quadratic
Medical treatments, proportion^*d*^	0.30^a^	0.17^b^	0.36^a^	0.25^ab^	0.05	0.038	0.840	0.787

^*a*^Data are least square means; *n* = 12 pens per treatment with 10 pigs per pen, totaling 480 pigs; 35-d growth experiment.

^*b*^NC: negative control, 0% ESBM; ESBM1: 7% ESBM; ESBM2: 14% ESBM; ESBM3: 21% ESBM. The inclusion of ESBM decreased by half in phase 2. Phase 3 was a common diet with no ESBM.

^*c*^Means within a row without a common superscript differ significantly (*P* < 0.05).

^*d*^Medical treatments were calculated as the total number of medical treatments administered per pen divided by the number of pigs allotted to pen.

**Table 5.** The effect of increasing ESBM on weekly fecal score^*a*,*b*,*c*,*d*^

						*P*-value^*e*^	
Item	NC	ESBM1	ESBM2	ESBM3	Pooled SEM	Diet	Diet × period
Fecal score^*f*^					0.2	0.003	0.409
Day 7	2.0	1.3	1.7	1.5			
Day 14	1.3	1.2	1.0	1.1			
Day 21	1.2	1.1	1.0	1.1			
Day 28	1.2	1.1	1.0	1.0			
Day 35	1.1	1.0	1.0	1.0			
Overall	1.4^a^	1.1^b^	1.2^b^	1.1^b^			

^*a*^Data are least square means; *n* = 12 pens per treatment with 10 pigs per pen, totaling 480 pigs; 35-d growth experiment

^*b*^NC: negative control, 0% ESBM; ESBM1: 7% ESBM; ESBM2: 14% ESBM; ESBM3: 21% ESBM. The inclusion of ESBM decreased by half in phase 2. Phase 3 was a common diet with no ESBM.

^*c*^Means within a row without a common superscript differ significantly (*P* < 0.05).

^*d*^ Dietary phases: phase 1 (days 0–14), phase 2 (days 14–28), and phase 3 (days 28–35).

^*e*^Period was significant for this variable (*P* < 0.001).

^*f*^Fecal scoring: 1 = solid, 2 = semisolid, 3 = semiliquid, 4 = liquid; pens were independently scored by two people and the score was averaged.

### Growth Performance

Overall, pigs fed NC and ESBM1 had greater final BW compared to ESBM2 and ESBM3 (diet, *P* = 0.001; Table 6). This pattern was also observed for overall ADG (diet, *P* = 0.001) and ADFI (diet, *P* = 0.001), but G:F was not impacted (*P* > 0.10). There was a decrease in final BW, overall ADG, and ADFI as the inclusion of ESBM increased (linear, *P* < 0.001).

**Table 6.** The effect of increasing ESBM on overall growth performance and feed efficiency of pigs^*a*,*b*,*c*,*d*^

						*P*-value		
Item	NC	ESBM1	ESBM2	ESBM3	SEM	Diet	Linear	Quadratic
Pens per treatment	12	12	12	12	–	–	–	–
Pigs per treatment, initial	120	120	120	120	–	–	–	–
Pigs per treatment, final^*e*^	106	108	106	106	–	–	–	–
Start BW (day 0)	6.3	6.3	6.4	6.3	0.01	0.324	0.712	0.141
Final BW (day 35)	20.0^a^	20.1^a^	19.0^b^	18.8^b^	0.3	0.001	<0.001	0.588
ADG, kg	0.38^a^	0.39^a^	0.35^b^	0.35^b^	0.01	0.001	<0.001	0.383
ADFI, kg	0.53^a^	0.54^a^	0.50^b^	0.50^b^	0.01	0.001	<0.001	0.646
G:F	0.71	0.71	0.71	0.70	0.004	0.302	0.108	0.323

^*a*^Data are least square means; *n* = 12 pens per treatment with 10 pigs per pen, totaling 480 pigs; 35-d growth experiment; growth calculations included pig days to account for removed pigs.

^*b*^NC: negative control, 0% ESBM; ESBM1: 7% ESBM; ESBM2: 14% ESBM; ESBM3: 21% ESBM. The inclusion of ESBM decreased by half in phase 2. Phase 3 was a common diet with no ESBM.

^*c*^Dietary phases: phase 1 (days 0–14), phase 2 (days 14–28), and phase 3 (days 28–35).

^*d*^Means within a row without a common superscript differ significantly (*P* < 0.05).

^*e*^12 pigs were removed from each treatment for necropsy on day 10.

In phase 1, NC and ESBM1 increased BW more than ESBM3 with ESBM2 being intermediate, but NC and ESBM1 improved BW compared to ESBM2 and ESBM3 in phases 2 and 3 (diet × period, *P* = 0.039; Table 7). Feeding NC increased G:F more than ESBM2 and ESBM3 in phase 1, while ESBM1 was intermediate; however, ESBM1 did increase G:F more than ESBM3 (diet × period, *P* = 0.008). In phase 2, ESBM1 increased G:F more than ESBM3 but not NC or ESBM2. The ESBM3 increased G:F compared to NC or ESBM1 in phase 3, with ESBM2 being intermediate. No diet × period interactions were observed for ADG or ADFI (*P* > 0.10).

**Table 7.** The effect of increasing ESBM on growth performance and feed efficiency of pigs by phase analyzed as a mixed model with a time-dependent variance structure^*a*,*b*,*c*,*d*^

						*P*-value^*e*^	
Item	NC	ESBM1	ESBM2	ESBM3	Pooled SEM	Diet	Diet × period
BW, kg					0.4	0.017	0.039
Day 0	6.3^a^	6.3^a^	6.4^a^	6.3^a^			
Day 14	9.1^b^	9.2^b^	8.9^bc^	8.6^c^			
Day 28	16.0^d^	15.9^d^	15.1^e^	14.7^e^			
Day 35	20.0^f^	20.1^f^	19.0^g^	18.8^g^			
ADG, kg					0.02	0.015	0.292
Days 0–14	0.20	0.21	0.18	0.17			
Days 14–28	0.48	0.47	0.43	0.42			
Days 28–35	0.58	0.59	0.56	0.59			
ADFI, kg					0.02	0.018	0.396
Days 0–14	0.26	0.26	0.24	0.22			
Days 14–28	0.67	0.67	0.62	0.61			
Days 28–35	0.89	0.88	0.84	0.86			
G:F					0.01	0.243	0.008
Days 0–14	0.82^a^	0.81^ab^	0.78^bc^	0.76^c^			
Days 14–28	0.71^de^	0.72^d^	0.71^de^	0.69^ef^			
Days 28–35	0.65^g^	0.65^g^	0.67^fg^	0.68^f^			

^*a*^Data are least square means; *n* = 12 pens per treatment with 10 pigs per pen, totaling 480 pigs; 35-d growth experiment; growth calculations included pig days to account for removed pigs.

^*b*^NC: negative control, 0% ESBM; ESBM1: 7% ESBM; ESBM2: 14% ESBM; ESBM3: 21% ESBM. The inclusion of ESBM decreased by half in phase 2. Phase 3 was a common diet with no ESBM.

Dietary phases: phase 1 (days 0–14), phase 2 (days 14–28), and phase 3 (days 28–35).

^*d*^Within a dependent variable, means without a common superscript (a–g) differ significantly (*P* < 0.05).

^*e*^Period was significant for all variables (*P* < 0.001).

### Fecal and Digesta Characteristics

Fecal DM increased as the inclusion of ESBM in the diets was increased (linear, *P* <0.001; Table 8). The WBC of ileal digesta was increased in pigs fed NC and ESBM3 compared to pigs fed ESBM1 and ESBM2 (quadratic, *P* = 0.058) but the diet did not affect the WBC of colonic digesta (*P* > 0.10). Furthermore, the diet did not impact the pH of ileal or colonic digesta (*P* > 0.10).

**Table 8.** The effect of increasing ESBM on fecal and digesta characteristics^*a*,*b*,*c*^

						*P*-value		
Item	NC	EBSM1	EBSM2	EBSM3	SEM	Diet	Linear	Quadratic
Fecal analyses								
DM, %	20.40^a^	20.91^a^	22.63^b^	24.62^c^	0.57	<0.001	<0.001	0.204
Ileal digesta analyses								
WBC, mL/g of dry digesta	1.39	0.99	0.73	1.37	0.27	0.237	0.797	0.058
pH	6.23	6.32	6.20	6.45	0.17	0.668	0.435	0.622
Colonic digesta analyses								
WBC, mL/g of dry digesta	0.23	0.21	0.15	0.19	0.05	0.701	0.437	0.599
pH	5.84	5.66	5.85	5.69	0.09	0.245	0.479	0.905

^*a*^Data are least square means; *n* = 12 replicates per treatment; fecal samples were collected from each pen on days 11–13; digesta samples were collected on day 10.

^*b*^NC: negative control, 0% ESBM; ESBM1: 7% ESBM; ESBM2: 14% ESBM; ESBM3: 21% ESBM

^*c*^Means within a row without a common superscript differ significantly (*P* < 0.05).

### Volatile Fatty Acids

In the ileum, increasing the inclusion of ESBM increased the concentration of acetate, butyrate, and total VFA (linear, *P* < 0.05; Table 9). Increasing ESBM linearly and quadratically increased propionate concentration in the ileum (linear, *P* = 0.006; quadratic, *P* = 0.003). The molar proportion (%) of ileal acetate was not affected by diet (*P* > 0.10), but the proportion of propionate was increased by NC and ESBM3 compared to ESBM1 and ESBM2 (quadratic, *P* = 0.029). The molar proportion of butyrate in the ileum was increased by ESBM3 compared to NC, ESBM1, and ESBM2 (diet, *P* = 0.023; linear, *P* = 0.019).

**Table 9.** The effect of increasing ESBM on VFA concentration and molar proportions in digesta^*a*,*b*,*c*^

						*P*-value		
Item	NC	ESBM1	ESBM2	ESBM3	SEM	Diet	Linear	Quadratic
Ileal digesta VFA, mM/L								
Acetate	3.27^a^	4.06^a^	4.50^ab^	6.98^b^	0.86	0.029	0.005	0.329
Propionate	0.10^a^	0.04^a^	0.08^a^	0.23^b^	0.03	0.002	0.006	0.003
Butyrate	0.02^a^	0.02^a^	0.02^a^	0.06^b^	0.01	0.029	0.015	0.071
Total	3.47	4.08	4.67	6.70	0.95	0.100	0.018	0.436
Ileal digesta VFA molar proportion, %								
Acetate	95.80	97.98	95.89	96.02	0.78	0.161	0.671	0.187
Propionate	3.48	1.29	2.15	3.53	0.80	0.146	0.773	0.029
Butyrate	0.53^a^	0.66^a^	0.52^a^	1.11^b^	0.15	0.023	0.019	0.137
Colonic digesta VFA, mM/L								
Acetate	74.73	82.58	69.75	80.81	9.05	0.717	0.892	0.857
Propionate	19.30	22.37	19.13	20.05	3.50	0.895	0.948	0.752
Butyrate	10.93	7.85	9.21	8.62	1.55	0.533	0.415	0.407
Total	103.08	109.31	96.77	103.05	11.85	0.897	0.808	0.998
Colonic digesta VFA molar proportion, %								
Acetate	73.04^ab^	75.64^a^	71.30^b^	76.97^a^	1.39	0.030	0.239	0.278
Propionate	18.96	17.68	19.11	16.10	0.93	0.103	0.097	0.362
Butyrate	8.58^ab^	6.57^a^	9.46^b^	6.61^a^	0.80	0.034	0.409	0.605

^*a*^Data are least square means; *n* = 12 replicates per treatment; ileal and colonic digesta samples were collected on day 10.

^*b*^NC: negative control, 0% ESBM; ESBM1: 7% ESBM; ESBM2: 14% ESBM; ESBM3: 21% ESBM.

^*c*^Means within a row without a common superscript differ significantly (*P* < 0.05).

Diet did not affect the concentration of acetate, propionate, butyrate, or total VFA in the colon (*P* > 0.10). Feeding ESBM1 and ESBM3 increased the molar proportion of acetate and decreased butyrate in the colon more than ESBM2 but not NC (diet, *P* < 0.05). The molar proportion of propionate in the colon tended to decrease as the inclusion of ESBM increased (linear, *P* = 0.097).

### Oxidative Status, Mucosal Cytokines, and LBP

The concentration of LBP in serum was not impacted by diet (*P* > 0.10; Table 10). The TAC of plasma was increased as the inclusion of ESBM increased (linear, *P* = 0.002). The MDA concentrations in plasma or ileal tissue were not impacted by diet (*P* > 0.10). There was no effect of diet on the following ileal mucosa cytokines: IFNγ, IL-1α, IL-1β, IL-2, IL-6, IL-8, IL-10, or IL-12 (*P* > 0.10). The concentration of TNF-α was not detectable in any of the samples. The concentration of IL-4 increased as the inclusion of ESBM increased (linear, *P* = 0.005). The NC diet tended to increase GM-CSF concentration compared to ESBM1 and ESBM2 but not ESBM3 (quadratic, *P* = 0.081). The ESBM2 diet tended to increase IL-1RA concentration compared to NC and ESBM1 with ESBM3 being intermediate (diet, *P* = 0.092). The concentration of IL-18 was increased by NC and ESBM2 compared to ESBM1 but not ESBM3 (diet, *P* = 0.098).

**Table 10.** The effect of increasing ESBM on LBP, markers of oxidative status, and mucosal cytokines^*a*,*b*,*c*^

						*P*-value		
Item	NC	ESBM1	ESBM2	ESBM3	SEM	Diet	Linear	Quadratic
Serum measures								
LBP, μg/mL	21.7	21.3	22.2	20.8	2.8	0.986	0.888	0.863
Plasma measures								
MDA, μM/μL	12.95	14.34	14.05	12.67	1.01	0.554	0.790	0.161
TAC, mM trolox	4.09^a^	4.11^a^	4.58^b^	4.49^b^	0.11	0.006	0.002	0.617
Ileal tissue measures								
MDA, μM/μg protein	0.63	0.62	0.68	0.52	0.05	0.194	0.276	0.149
Ileal mucosa measures, ng/g								
GM-CSF	0.61	0.39	0.44	0.48	0.07	0.175	0.282	0.081
IFNγ	20.33	19.08	19.01	18.98	1.46	0.895	0.532	0.680
IL-1α	1.75	1.39	1.59	1.56	0.15	0.383	0.551	0.260
IL-1β	18.01	15.86	16.18	16.45	2.96	0.941	0.715	0.658
IL-1RA	8.46	8.43	11.61	9.26	0.98	0.092	0.213	0.244
IL-2	1.33	1.20	1.29	1.31	0.10	0.802	0.939	0.472
IL-4	0.63^a^	0.65^a^	1.17^b^	1.01^b^	0.12	0.009	0.005	0.484
IL-6	2.34	2.21	2.26	2.22	0.07	0.568	0.343	0.542
IL-8	491.27	394.24	426.54	448.03	45.11	0.447	0.622	0.180
IL-10	0.41	0.36	0.42	0.40	0.04	0.764	0.883	0.725
IL-12	4.75	3.73	4.64	4.34	0.58	0.605	0.908	0.537
IL-18	180.04	138.52	180.88	167.79	13.12	0.098	0.924	0.286

^*a*^Data are least square means; *n* = 12 replicates per treatment; all tissue and blood samples were collected on day 10.

^*b*^NC: negative control, 0% ESBM; ESBM1: 7% ESBM; ESBM2: 14% ESBM; ESBM3: 21% ESBM

^*c*^Means within a row without a common superscript differ significantly (*P* < 0.05).

### Ileal Tissue Gene Transcription

Ileal mRNA abundance of *claudin*-*3* (*CLDN3*) and *CLDN4* was not altered by diet (*P* > 0.10; Table 11). Feeding ESBM2 increased the abundance of *occludin* (*OCLN*) compared to ESBM3, with NC and ESBM1 being intermediate (quadratic, *P* = 0.049). The abundance of *zonula-occludens-1* (*ZO-1*) was increased by ESBM1 compared to NC and ESBM3 but not ESBM2 (quadratic, *P* = 0.013).

**Table 11.** The effect of increasing ESBM on relative ileal gene mRNA abundance^*a*,*b*,*c*^

						*P*-value		
Gene	NC	ESBM1	ESBM2	ESBM3	SEM	Diet	Linear	Quadratic
*CLDN3*	1.20	1.69	1.25	0.84	0.45	0.679	0.538	0.570
*CLDN4*	1.13	1.65	1.15	0.98	0.29	0.313	0.429	0.188
*OCLN*	1.59	2.22	2.44	0.87	0.59	0.191	0.369	0.049
*ZO-1*	1.17	1.86	1.58	1.13	0.29	0.091	0.771	0.013

^*a*^Data are least square means; *n* = 12 replicates per treatment; ileal tissue samples were collected on day 10.

^*b*^NC: negative control, 0% ESBM; EBSM1: 7% ESBM; ESBM2: 14% ESBM; ESBM3: 21% ESBM

^*c*^Means within a row without a common superscript differ significantly (*P* < 0.05).

### Gut Morphology

Villus height in the ileum was not impacted by diet (*P* > 0.10; Table 12). However, crypt depth was slightly decreased by increasing the inclusion of ESBM3 (linear, *P* = 0.069). No difference in the V:C ratio was observed (*P* > 0.10).

**Table 12.** The effect of increasing ESBM on ileal morphology^*a*,*b*,*c*^

						*P*-value		
Item	NC	ESBM1	ESBM2	ESBM3	SEM	Diet	Linear	Quadratic
Villus height, µm	342.7	365.2	341.8	322.1	15.5	0.294	0.228	0.182
Crypt depth, µm	244.8	234.1	242.4	222.6	6.9	0.120	0.069	0.517
Villi height:crypt depth	1.52	1.67	1.49	1.51	0.08	0.387	0.598	0.431

^*a*^Data are least square means; *n* = 12 replicates per treatment; ileal tissue samples were collected on day 10 and fixed in 10% neutral-buffered formalin for 48 h followed by 70% ethanol before histology slides were made.

^*b*^NC: negative control, 0% ESBM; ESBM1: 7% ESBM; ESBM2: 14% ESBM; ESBM3: 21% ESBM.

^*c*^Means within a row without a common superscript differ significantly (*P* < 0.05).

## DISCUSSION

Overall, feeding increasing levels of ESBM decreased the final BW, ADG, and ADFI in a linear fashion but had no impact on feed efficiency. The BW of pigs was decreased by feeding 21% ESBM during phase 1 or feeding greater than 7% ESBM in phase 2. Jones et al. (2018a) reported results similar to our study, as feeding increasing levels of ESBM (6.7–20% ESBM in phase 1; 5–15% ESBM in phase 2) linearly decreased final BW, ADG, and ADFI. Furthermore, 15% ESBM has been shown to decrease ADG and ADFI compared to the control diet, but feed efficiency was improved (Jones et al., 2018b). Contrary to our results, Zhu et al. (1998) and Zhou et al. (2011) both reported improved performance by feeding 3.5%, 7%, and 10.5% or 5%, 10%, and 15% ESBM, respectively. Other studies have reported increased ADG and G:F by feeding 9% ESBM and decreasing SBM (Ma et al., 2019a, 2019b). These growth improvements are attributed to the increased nutrient digestibility and the reduced concentration of ANF in ESBM (Zhou et al., 2011).

In the current experiment, the reduced growth rate and feed intake that resulted from feeding increased ESBM were unexpected, but there are possible explanations. Increasing the inclusion of ESBM did not impact feed efficiency, suggesting that the decrease in ADG was due to reduced ADFI. Reduced feed intake could be attributed to the increased WHC of diets as the inclusion of ESBM increased. The WHC is a measure of a feedstuff’s ability to hold water within its matrix (Giger-Reverdin, 2000). As feed particles move through the GIT and absorb water, the feed can swell and limit consumption (Anguita et al., 2007). It has been shown that the ADFI of pigs decreases as the WHC of feed increases (Kyriazakis and Emmans, 1995; Ndou et al., 2013). Furthermore, Zhang et al. (2001) reported that the transit rate of digesta decreased in diets containing 19% ESBM compared to diets with 23.5% SBM or 19% ESBM plus 1% stachyose. Feed intake has been inversely linked to the transit rate of digesta through the GIT, so feeding ESBM could slow this transit rate and limit the feed intake of pigs (Ratanpaul et al., 2019).

We observed improvements in overall fecal score and fecal DM when ESBM was included in the diet, indicating that the ESBM could reduce diarrhea in weaned pigs. The ESBM1 diet also reduced the number of medical treatments required, but higher levels provided no benefit. Ma et al. (2019b) reported that feeding ESBM reduced the incidence of diarrhea in weaned pigs compared to conventional SBM. These results are likely due to the reduced concentration of antigenic proteins and NDO in ESBM. The hypersensitivity associated with glycinin and β-conglycinin in SBM has been linked to malabsorption of nutrients and diarrhea in young pigs (Zhang et al., 2003; Sun et al., 2008b). Furthermore, pigs do not possess the endogenous enzymes that are needed to digest the galactooligosaccharides stachyose and raffinose (Dersjant-Li and Peisker, 2010). Makinde et al. (1996) hypothesized that decreased digestion and absorption of soybean carbohydrates in the small intestine would increase fermentation in the colon, affecting the osmolarity of the colonic contents and decreasing water absorption (Grahofer et al., 2016).

Although the NDO in SBM could contribute to increased diarrhea, it has been suggested that the NDO could also act as a prebiotic in the GIT of growing pigs (Smiricky-Tjardes et al., 2003). Undigested carbohydrates can be fermented by bacteria in the GIT, producing VFAs that serve as an energy source for the host. The majority of microbial fermentation occurs in the cecum and colon, but limited fermentation takes place in the small intestine as well (Choct et al., 2010). Cervantes-Pahm and Stein (2010) reported that SBM has increased concentrations of stachyose and raffinose compared to ESBM. However, our results show a linear increase in acetate, butyrate, propionate, and total VFA concentration in ileal digesta associated with an increase in the inclusion level of ESBM and a reduction in the SBM content. Therefore, increasing the inclusion of ESBM may slow the digesta transit rate, giving microbes in the ileum more time to ferment NDO and other nonstarch polysaccharides to produce VFAs (Zhang et al., 2001; Zhang et al., 2003). In contrast, ESBM inclusion did not impact the colonic concentration of individual or total VFAs compared to SBM. The pH of colonic digesta was not changed by ESBM, which can be explained by the lack of differences in VFA concentration. Zhang et al. (2003) observed no differences in total VFA production in the ileum or colon when feeding diets with 0% SBM, 0% SBM plus 1% stachyose, or 20% SBM, indicating that the concentration of NDO in a complete corn-SBM diet is not high enough to have a prebiotic effect.

Weaning can cause oxidative stress due to an imbalance between the production of antioxidants and scavenging of reactive oxygen species (ROS), potentially reducing the activity of antioxidant enzymes and causing damage to DNA, lipids, or protein (Betteridge, 2000; Yin et al., 2014). Malondialdehyde is a product of lipid peroxidation that indicates oxidative damage to lipids by ROS (Del Rio et al., 2005). Total antioxidant capacity represents the ability of endogenous and dietary antioxidants to prevent oxidative damage (Ghiselli et al., 2000). We observed no change in MDA concentration in plasma or ileal tissue when feeding ESBM; however, the TAC of plasma linearly increased as the inclusion of ESBM increased and SBM decreased. Feeding ESBM has been shown to reduce MDA concentration and improve TAC in the serum of weaned pigs (Ma et al., 2019a, 2019b). It was also reported that ESBM increased serum superoxide dismutase and glutathione peroxidase activity, indicating that feeding ESBM may alleviate oxidative stress and improve TAC by increasing antioxidant enzyme activity (Betteridge, 2000; Ma et al., 2019a, 2019b).

The production of ROS and subsequent oxidative stress have also been linked to the immune response (Lugrin et al., 2014). The production of cytokines to signal and modulate an inflammatory response is a crucial step in activating the GIT immune system (Pié et al., 2004). In our study, there was a tendency for the ESBM treatments to decrease the ileal mucosal concentration of GM-CSF. The GM-CSF has a pro-inflammatory role and activates ROS-producing neutrophils, further explaining the modulation of oxidative stress by ESBM (Shiomi et al., 2016). The mucosal concentration of IL-4 was linearly increased as the inclusion of ESBM increased. The IL-4 is an anti-inflammatory cytokine that helps regulate the hypersensitivity reaction caused by glycinin and β-conglycinin by stimulating the production of immunoglobulin E and differentiation of type 2 helper T-cells (Sun et al., 2008a). Multiple studies have reported increases in serum or mucosal IL-4 concentration when pigs are fed purified glycinin, making our results unexpected because the concentration of glycinin is reduced in ESBM (Sun et al., 2008a; Wu et al., 2016).

The intestinal epithelial barrier acts in a defensive role to prevent antigens, pathogens, and toxins from translocating through the lumen into the body (Awad et al., 2017). The paracellular permeability of the epithelium is maintained by transmembrane tight junction (TJ) proteins. A decrease in the mRNA abundance of TJ proteins, which typically occurs during weaning or an immune response, can indicate the disruption of TJ protein complexes and increased permeability of the epithelial barrier (Hu et al., 2013). In our study, the highest mRNA abundance of *OCLN* or *ZO-1* was observed in the ESBM1 or ESBM2 treatments compared to NC or ESBM3, resulting in a quadratic response. These improvements in TJ protein mRNA abundance are likely due to the reduced concentration of glycinin and β-conglycinin in ESBM. However, the reduced mRNA abundance of *OCLN* and *ZO-1* after feeding the ESBM3 diet was unexpected and cannot be explained by this study. The ESBM3 diet should have contained the lowest levels of glycinin and β-conglycinin, resulting in an improved mRNA abundance of TJ proteins compared to NC. Similar to our study, Ma et al. (2019b) reported increased protein abundance of *OCLN* and *ZO-1* after feeding 9% ESBM. Furthermore, Zhao et al. (2014) reported decreases in the mRNA abundance of *OCLN* and *ZO-1* when intestinal porcine epithelial cells were treated with glycinin or β-conglycinin. The mechanisms causing this reduction are largely unknown but may be linked to the increased apoptosis of enterocytes when antigenic proteins are fed (Bojarski et al., 2004; Zhao et al., 2010).

The main epithelial cells lining the villi in the small intestine are absorptive enterocytes, so villus atrophy results in less surface area for nutrient absorption to occur (Yang and Liao, 2019). Villus atrophy without crypt hyperplasia typically occurs after weaning due to decreased feed intake and slowed production of crypt cells (Pluske et al., 1997). However, the antigenic proteins in SBM can cause villus atrophy and crypt hyperplasia, indicating an increased rate of cell loss on the villi (Li et al., 1991; Pluske et al., 1997). Improvements in duodenal morphology after feeding ESBM have been reported, but the changes in ileal morphology have been inconsistent (Ma et al., 2019a, 2019b). Though our results showed no impact of ESBM on villus height or V:C in the ileum, there was a tendency for increasing ESBM to reduce crypt depth. Feed intake decreased as the inclusion of ESBM increased, possibly resulting in villus atrophy without crypt hyperplasia. Therefore, the antigenic proteins in SBM may have impacted villus height but treatment differences could not be differentiated.

In conclusion, the pigs that were fed the two highest levels of ESBM (14% or 21% in phase 1; 7% or 10.5% in phase 2) had decreased overall BW, ADG, and ADFI compared to pigs fed the control and lowest ESBM diets. However, the inclusion of ESBM did not have an impact on the overall feed efficiency of pigs. Our hypothesis was partially incorrect, as increasing the inclusion of ESBM linearly decreased growth performance rather than improving it. This response may be driven by reductions in feed intake due to increased WHC of ESBM diets. However, the inclusion of ESBM did beneficially modulate markers of oxidative stress and intestinal health and function. Feeding ESBM improved overall fecal score and increased fecal DM, indicating that ESBM could reduce diarrhea in weaned pigs. Furthermore, the ESBM appeared to increase the ileal fermentation of carbohydrates due to increased VFA production, but this response did not occur in the colon. Oxidative status and intestinal barrier integrity were improved by ESBM, but the impact on intestinal inflammation and morphology was minimal. This research identified key aspects of intestinal health and function that may be improved by replacing portions of SBM with ESBM. However, further research is needed to determine the ideal inclusion level of ESBM to optimize growth performance while benefiting various aspects of GIT physiology and function.
